# Relationship between device‐detected subclinical atrial fibrillation and heart failure in patients with cardiac resynchronization therapy defibrillator

**DOI:** 10.1002/clc.23471

**Published:** 2020-09-29

**Authors:** Shuhei Arai, Mitsuharu Kawamura, Toshihiko Gokan, Kosuke Yoshikawa, Ko Ogawa, Akinori Ochi, Yumi Munetsugu, Hiroyuki Ito, Toshiro Shinke

**Affiliations:** ^1^ Division of Cardiology Showa University School of Medicine Tokyo Japan

**Keywords:** cardiac resynchronization therapy, heart failure, inappropriate therapy, subclinical atrial fibrillation

## Abstract

**Background:**

Atrial fibrillation (AF) is a leading preventable cause of heart failure (HF) for which early detection and treatment is critical. Subclinical‐AF is likely to go untreated in the routine care of patients with cardiac resynchronization therapy defibrillator (CRT‐D).

**Hypothesis:**

The hypothesis of our study is that subclinical‐AF is associated with HF hospitalization and increasing an inappropriate therapy.

**Methods:**

We investigated 153 patients with an ejection fraction less than 35%. We divided into three groups, subclinical‐AF (n = 30), clinical‐AF (n = 45) and no‐AF (n = 78). We compared the baseline characteristics, HF hospitalization, and device therapy among three groups. The follow‐up period was 50 months after classification of the groups.

**Results:**

The average age was 66 ± 15 years and the average ejection fraction was 26 ± 8%. Inappropriate therapy and biventricular pacing were significantly different between subclinical‐AF and other groups (inappropriate therapy: subclinical‐AF 13% vs clinical‐AF 8.9% vs no‐AF 7.7%: *P* = .04, biventricular pacing: subclinical‐AF 81% vs clinical‐AF 85% vs no‐AF 94%, *P* = .001). Using Kaplan‐Meier method, subclinical‐AF group had a significantly higher HF hospitalization rate as compared with other groups. (subclinical‐AF 70% vs clinical‐AF 49% vs no‐AF 38%, log‐rank: *P* = .03). In multivariable analysis, subclinical‐AF was a predictor of HF hospitalization.

**Conclusions:**

Subclinical‐AF after CRT‐D implantation was associated with a significantly increased risk of HF hospitalization. The loss of the biventricular pacing and increasing an inappropriate therapy might affect the risk of HF hospitalization.

## 1 INTRODUCTION

Cardiac resynchronization therapy with defibrillator (CRT‐D) is an approved treatment for patients with an advanced staged of heart failure (HF) in sinus rhythm (SR) with low left ventricular ejection fraction (LVEF) and ventricular dyssynchrony. This therapy is associated with a reduction in symptoms, improvement in LVEF, and decrease in hospitalization and mortality.^1^, ^2^, ^3^ However, the appropriate use of CRT is not well defined in patients with atrial fibrillation (AF). CRT is not as effective in patients with AF because of inadequate biventricular capture and loss of atrioventricular (AV) synchrony.^4^, ^5^, ^6^ A recent study^7^ showed that ablation of the AV junction ablation was associated with a significantly reduced likelihood and rate of AF‐related hospitalization, irrespective of whether a right ventricular (RV) or a biventricular pacemaker was implanted. In patients who underwent ablation of the AV junction, implantation of a biventricular pacemaker was associated with a 38% reduction in the rate of HF hospitalizations as compared with patients who had an RV pacemaker. AF might be symptomatic and asymptomatic, or both.^8^, ^9^ Therefore, HF or stroke can be the first clinical manifestation of asymptomatic AF. The Assert study^10^, ^11^ showed that subclinical‐AF (S‐AF) was associated with stoke event. However, these studies found no relationship between S‐AF and HF hospitalization. Furthermore, the relationship between S‐AF and HF hospitalization is still unclear, and S‐AF is likely to go untreated in patients with CRT‐D. The purpose of this study was to investigate the relationship between S‐AF and HF hospitalization in patients with cardiac dysfunction using CRT‐D.

## 2 METHODS

### 2.1 Patients and study protocol

This study was a retrospective analysis. Among 242 consecutive patients with CRT‐D implantation, 153 consecutive patients without history of AF were included in this study between November 2006 and July 2016 at our hospital. The eligibility criteria for CRT‐D implantation included advanced HF, a decreased LVEF (less than 35%), and a wide QRS complex (> 120 ms). We divided the patients into three groups, S‐AF group, clinical‐AF (C‐AF) group, and no‐AF group. We defined a CRT responder as a patient with a reduced LV end‐diastolic volume of >15% at the 6‐month follow‐up as compared with baseline. AF was defined as atrial tachycardia that continued more than 6 minutes and an atrial rate reached 190 beats per minute. Persistent AF was defined as non‐self‐terminating AF lasting ≧7 days and requiring pharmacologic or electrical conversion to restore SR. Paroxysmal AF was defined as self‐terminating AF < 7 days. No patient had permanent AF, which was defined as sustained arrhythmia despite cardioversion in this study. S‐AF was defined as AF detected by a device report without symptoms. We defined C‐AF as the presence of symptoms, such as palpitation, chest oppression, dizziness, and dyspnea, and AF as detected by a device report or ECG after CRT‐D implantation. Patients were assessed during the first 3 months after CRT‐D implantation excluding the 2 weeks as the blanking period. The follow‐up period was 50 months after classification of the groups. We investigated HF hospitalization and stroke. Furthermore, we investigated appropriate therapy or inappropriate therapy by device report during follow‐up period. We further divided the patients into two groups: Patients who required hospitalization for HF during follow‐up period (HF group) and patients who did not require hospitalization for HF during follow‐up period (no‐HF group). Patients were excluded from the study if: Patients were not eligible for enrollment if (a) patients had a history of C‐AF or atrial flutter on electrocardiography (ECG) or cardiac monitor before CRT‐D implantation, (b) they underwent cardiac surgery within 1 month after implantation, (c) We also excluded no‐AF patients develop AF during the 50‐month follow‐up. All patients gave written informed consent before device implantation. This study was approved by the institutional committee at our institution.

### 2.2 Measurements

We evaluated the patients' baseline clinical characteristics using data obtained from the electronic medical records, the telephone contact for the patient's family, and the device report. We also examined the records to determine HF hospitalization, stroke, and cardiac event. In addition, we evaluated implantable cardioverter defibrillator (ICD) therapies from the device reports, including shock therapy and anti‐tachycardia pacing (ATP) therapy. ATP was attempted with eight pulses at 88% of the measured cycle length with a 10‐ms decrement between bursts. The initial device shock was attempted at the attending physician's discretion. The remaining device shock consisted of the maximal energy shocks. The ICD was programmed at the attending physician's discretion. An appropriate therapy event was defined as ATP and shock therapies delivered for ventricular tachycardia and ventricular fibrillation. An inappropriate therapy event was defined as ATP and shock therapies delivered for tachycardia including AF, supraventricular tachycardias, or sinus tachycardia, and device error. The EF was assessed with the biplane Simpson's equation using the apical 4‐chamber and 2‐chamber views.

### 2.3 Statistical analysis

Data are presented as mean ± SD. Multiple‐group comparisons were obtained by analysis of variance. Categorical data are summarized as frequencies and percentages. Differences in baseline characteristics among patients with S‐AF group, C‐AF group, and no‐AF group were analyzed using unpaired Student *t* tests. Differences in baseline characteristics among patients with HF hospitalization and without HF hospitalization were analyzed using unpaired Student *t* tests. The paired Student *t* test was used to compare continuous data within the subgroups during follow‐up. The Kaplan‐Meier method was used to analyze the time to the occurrence of the therapy event and HF hospitalization during the follow‐up period, which was compared using the log‐rank test. The hazard ratio and its confidence intervals were estimated using the Cox regression model. *P* values <.05 were considered statistically significant. All statistical analyses were performed with the JMP 14 software (SAS Institute, Inc., Cary, North Carolina). The authors had full access to and take full responsibility for the integrity of the data. All authors have read and agree to the manuscript as written.

## 3 RESULTS

### 3.1 Patient characteristics

We investigated and analyzed a total of 153 patients with a CRT‐D. In our study, S‐AF group included 30 (19%) patients, C‐AF group included 45 (29%) patients, and no‐AF group included 78 (52%) patients. Table 1 shows the baseline characteristics of the patients among three groups. Patients in S‐AF group were significantly older than those with in no‐AF group (69 ± 22 years vs 64 ± 15 years, *P* = .04). The detection of the first episode with S‐AF group was significantly longer as compared to those with C‐AF group (74 ± 58 day vs 44 ± 40 day, *P* = .03). Biventricular pacing with S‐AF group was significantly lower as compared to those with no‐AF group (subclinical‐AF 81% vs clinical‐AF 85% vs no‐AF 94%, *P* = .001). There was no significant difference with catheter ablation for AF; S‐AF group 1(3.3%) vs C‐AF group 3 (6.6%). There were no significant differences with drug including beta blockers, ACE‐I/ARB, Ca antagonist, diuretics, digoxin, and amiodarone among three groups.

**TABLE 1:** Baseline characteristics among three groups

	Subclinical AF (n = 30)	Clinical AF (n = 45)	No‐AF (n = 78)	*P* value
Age (year)	69 ± 22	66 ± 17	64 ± 15	.04
Male sex ‐ no. (%)	22 (73%)	32 (71%)	60 (76%)	.35
Body mass index	23 ± 9	22 ± 8	23 ± 7	.68
CHADS2 score	2.2 ± 1.2	2.3 ± 1.3	2.2 ± 1.2	.65
Underlying disease ‐no.(%)				
Hypertension	15 (50%)	23 (51%)	44 (56%)	.42
Diabetes mellitus	10 (33%)	14 (31%)	28 (35%)	.43
Prior stroke	2 (7%)	5 (11%)	6 (9%)	.53
Prior myocardial infarction	7 (23%)	13 (29%)	15 (19%)	.27
Ischemic cardiomyopathy	13 (43%)	19 (43%)	26 (33%)	.16
Non‐ischemic cardiomyopathy	17 (57%)	26 (57%)	54 (67%)	.22
Paroxysmal AF	18 (60%)	16 (36%)		.15
Persistent AF	12 (40%)	29 (64%)		.12
AF burden (%)	36 ± 22	41 ± 25		.25
Time to detection of the first event	74 ± 58 day	44 ± 40 day		.03
Primary prevention (ICD)	20 (67%)	32 (71%)	47 (60%)	.35
Secondary prevention (ICD)	10 (33%)	13 (29%)	31 (40%)	.38
Ejection fraction (%)	26 ± 22	25 ± 18	27 ± 14	.31
Left atrial size (mm)	40 ± 15	41 ± 20	37 ± 18	.08
CRT responder‐no. (%)	24 (80%)	36 (80%)	62 (79%)	.56
PVC burden/24 hr	0.5 ± 0.3	0.6 ± 0.4	0.4 ± 0.6	.45
Biventricular pacing (%)	81 ± 13	85 ± 6	94 ± 7	.001
Mode switch (number of times)	18 ± 11	10 ± 6	0.2 ± 0.7	.001
Catheter ablation for AF^a^	1 (3.3%)	3 (6.6%)		.22
Medication‐ no. (%)				
Beta‐blocker	24 (80%)	38 (84%)	60 (77%)	.15
ACE‐I/ARB	18 (60%)	29 (64%)	52 (66%)	.45
Ca antagonist	3 (10%)	5 (11%)	7 (9%)	.45
Diuretics	27 (90%)	41 (91%)	74 (95%)	.32
Digoxin	3 (10%)	6 (13%)	7 (9%)	.41
Amiodarone	4 (13%)	7 (16%)	13 (16%)	.52

Abbreviations: ACE, angiotensin converting enzyme; AF, atrial fibrillation; ARB, angiotensin II receptor blocker; CRT, cardiac resynchronization therapy; ICD, implantable cardioverter defibrillator.

^a^ Catheter ablation for AF was performed after device implant.

### 3.2 Clinical outcomes

**FIGURE 1:**
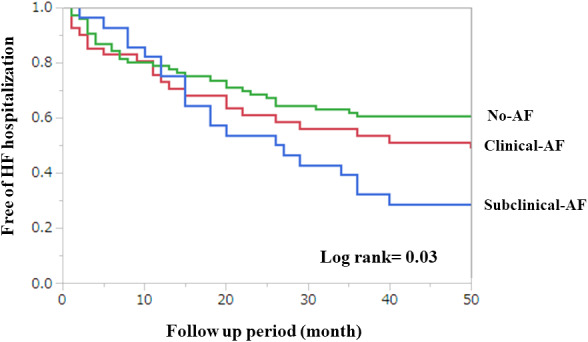
Kaplan–Meier curve for heart failure hospitalization. Kaplan–Meier estimates of the percentage of patients remaining free from heart failure hospitalization among three groups. The x‐axis shows the number of days of follow‐up after cardiac resynchronization therapy defibrillator implantation

Table 2 shows the clinical outcomes after device implantation. Patients in S‐AF group had a higher rate of HF hospitalization as compared with C‐AF group and no‐AF group (70% vs 49% vs 38%, *P* = .03). Patients with S‐AF had a 2‐fold higher rate of HF hospitalization as compared with no‐AF patients. S‐AF group presented with inappropriate therapy more frequently as compared with no‐AF group (13% vs 7.7%, *P* = .04). There were no significant differences with stroke or myocardial infarction among three groups. Figure 1 shows the Kaplan–Meier curve for HF hospitalization among three groups. HF with S‐AF group had a significantly higher prevalence of HF as compared with C‐AF and no‐AF group (*P* = .03 by log‐rank).

**TABLE 2:** Clinical outcomes after device implantation

	Subclinical AF	Clinical AF	No‐AF	*P* value
No.%	30	45	78	
Heart failure‐no, (%)	21 (70%)	22 (49%)	30 (38%)	.03
Stroke/TIA‐no,(%)	5 (17%)	5 (11%)	9 (12%)	.18
Myocardial infarction‐no,(%)	2 (6.7%)	3 (6.7%)	5 (6.4%)	.73
Device therapy‐no,(%)				
Appropriate therapy	3 (10%)	3 (8.8%)	11 (14%)	.08
Inappropriate therapy	4 (13%)	4 (8.9%)	6 (7.7%)	.04

*Note*: Appropriate therapy, shock and/or anti‐tachycardia pacing due to ventricular arrhythmias; inappropriate therapy, shock and/or anti‐tachycardia pacing due to AF/AT or sinus tachycardia.

Abbreviation: AF, atrial fibrillation.

### 3.3 Number of appropriate and inappropriate ICD therapies

In S‐AF group (n = 30), 3 (10%) patients had appropriate therapies and 4 (13%) patients had inappropriate therapies. In C‐AF group (n = 45), 3 (8.8%) patients had appropriate therapies and 4 (8.9%) patients had inappropriate therapies. In no‐AF group (n = 78), 11 (14%) patients had appropriate therapies and 6 (7.7%) patients had inappropriate therapies. Patients in no‐AF group tended to present with appropriate therapies more than in C‐AF group.

### 3.4 Predictors of HF

Table 3 presented a comparison of the baseline characteristics between HF group and no‐HF group. Cardiac rehabilitation without HF group tended to be more frequently as compared to those with HF group (35% vs 27%, *P* = .08). Patients with HF presented with S‐AF more frequently as compared in patients without HF (28% vs 11%, *P* = .009). The biventricular pacing in HF group was significantly lower than in no‐HF group (84% vs 92%, *P* = .01). The mode switch rate in HF group was significantly higher than in no‐HF group (8.1 times vs 4.3 times, *P* = .03). In the multivariate analysis (Table 4), when comparing HF group and no‐HF group, the independent predictor for HF hospitalization was presence of S‐AF (hazard ratio; 4.47, confidence interval; 1.43‐7.52, *P* = .01).

**TABLE 3:** Baseline characteristics of patients with and without heart failure after device implantation

	Heart failure (+)	Heart failure (−)	*P* value
Number	73	80	
Age (year)	66 ± 12	65 ± 15	.82
Male sex – no. (%)	53 (73%)	59 (74%)	.84
Body mass index	24 ± 11	21 ± 8	.22
CHADS2 score	2.3 ± 1.5	2.1 ± 1.4	.32
Underlying disease ‐no. (%)			
Hypertension	40 (54%)	41 (51%)	.68
Diabetes mellitus	26 (35%)	26 (32%)	.43
Hyperlipidemia	47 (64%)	42 (52%)	.06
Chronic kidney disease	35 (48%)	39 (49%)	.88
Ischemic cardiomyopathy	27 (37%)	31 (39%)	.58
Ejection fraction (%)	25 ± 5	28 ± 9	.12
Cardiac rehabilitation	20 (27%)	28 (35%)	.08
Subclinical AF	21 (28%)	9 (11%)	.009
Device therapy			
Appropriate therapy ‐no. (%)	8 (11%)	9 (11%)	.82
Inappropriate therapy ‐no. (%)	7 (10%)	7 (9%)	.42
CRT Responder ‐no. (%)	62 (85%)	60 (75%)	.26
PVC burden/24‐hours	0.6 ± 0.5	0.4 ± 0.3	.52
Biventricular pacing (%)	84%	92%	.01
Mode switch	8.1 ± 10	4.3 ± 4	.03
Catheter ablation for AF^a^	2 (2.7%)	2 (2.5%)	.72
Medication‐ no. (%)			
Beta‐blocker	60 (82%)	62 (78%)	.88
ACE‐I/ARB	45 (61%)	54 (67%)	.12
Ca antagonist	7 (10%)	8 (10%)	.82
Diuretics	69 (95%)	73 (91%)	.22
Digoxin	7 (10%)	9 (11%)	.56
Amiodarone	10 (14%)	14 (18%)	.35

Abbreviations: ACE, angiotensin converting enzyme; AF, atrial fibrillation; ARB, angiotensin II receptor blocker; CRT, cardiac resynchronization therapy.

^a^ Catheter ablation for AF was performed after device implant.

**TABLE 4:** Multivariate analysis of heart failure

Variable	HR	CI	*P* value
Ejection fraction (<30%)	0.65	0.31‐1.35	.32
Cardiac rehabilitation	1.97	0.95‐4.05	.09
Hyperlipidemia	1.51	0.88‐2.74	.25
ARB/ACE‐I	0.66	0.35‐1.11	.12
Subclinical‐AF	4.47	1.43‐13.9	.01
Biventricular pacing (< 85%)	1.95	0.66‐2.56	.08
Mode switch/3 month (> 10)	0.61	0.22‐1.59	.31

Abbreviations: ACE, angiotensin converting enzyme; AF, atrial fibrillation; ARB, angiotensin II receptor blocker; CRT, cardiac resynchronization therapy.

## 4 DISCUSSION

### 4.1 Main findings

This study showed the relationship between S‐AF and HF hospitalization in patients with CRT‐D implantation. S‐AF was found to be an independent predictor for HF hospitalization. Patients with S‐AF presented with inappropriate therapy more frequently as compared to those with no‐AF group.

### 4.2 Detection of S‐AF and relationship between S‐AF and stroke

Previous studies have reported that the rate of device‐detected S‐AF is about 10%^10^, ^11^. In our study, the rate of device‐reported S‐AF was high (19.6%), because all patients had a low EF with CRT‐D and the follow‐up period was long. Mahajan R^12^ et al reported the figure of seven studies consisting of a total of 15 353 patients, and there was a significant association between S‐AF and stroke, with an average odds ratio of 2.41. However, there was no significant relationship between S‐AF and stroke event, because our study was small with only 153 patients included.

### 4.3 Relationship between S‐AF and HF hospitalization

The ASSERT study^10^ did not find a significant relationship between S‐AF and HF. In that study, patients had a pacemaker or ICD. On the contrary, the result of our study indicated a significant relationship between S‐AF and HF hospitalization. Because patients in our study had a low EF and all patients had CRT‐D. Furthermore, a decrease biventricular pacing might affect for HF hospitalization. Nakajima^13^ et al described a significant proportion of the patients developed HF due to an AF episode itself, even among CRT responders. Once AF occurred, the biventricular pacing decreased significantly, and the patients who had a lower biventricular pacing during periods of AF exhibited a worse clinical outcome.

Furthermore, patients with AF and a biventricular pacing <90% had a higher incidence of HF or death than both the patients with an AF and biventricular pacing ≥90% and those with SR.

Another study^14^ reported that AF in CRT patients was associated with an increase in HF hospitalization and death, mainly because uncontrolled ventricular rates reduce the delivery of an optimal of biventricular pacing. A biventricular pacing <90% was associated with a higher incidence of HF and death, and a biventricular pacing >98% significantly reduced HF and death. In our study, the biventricular pacing in the S‐AF group was significantly lower as compared with no‐AF group (81% vs 94%). Previous studies have described AF patients as S‐AF with C‐AF, whereas our study divided patients into the S‐AF or C‐AF group. This classification is important for HF in patients receiving CRT‐D. In patients with C‐AF, the ventricular rate can be controlled and the SR returned using medication. On the other hand, patients with S‐AF had no symptoms and time to the detection of the first episode was late.

Therefore, the biventricular pacing in the S‐AF group was low and the decrease of biventricular pacing might affect an increased incidence of HF hospitalization. Frequent PVCs might affect the biventricular pacing and might affect HF hospitalization. However, there was no significant difference with PVC burden by 24 hours monitoring among three groups. In this study, data was evaluated after CRT‐D implant. Therefore, PVC burden had little effect on the biventricular pacing. It appeared that patients with S‐AF had less biventricular pacing due to AF. One potential of the mechanism underling AF progression with HF might be the inability of patients predisposed to HF to tolerate prolonged, rapid ventricular rates during S‐AF, leading to the clinical unmasking of HF. Furthermore, tachycardia induced cardiomyopathy due to prolonged episodes of S‐AF may be an important factor in some patients.^15^ Atrial systole constitutes a considerable proportion of the cardiac output in patients predisposed to HF, and its loss during episodes of S‐AF might also account for some of the observed increase in HF risk.^16^

Nishinarita^17^ et al reported that in patients without clinical AF who had a cardiac device, new‐onset atrial high‐rate episode identified as asymptomatic AF was detected in 32.7% of the patients during the first year after implantation of the cardiac device. Furthermore, a higher atrial high‐rate episode burden was more strongly associated with future risk of worsening HF in patients with a cardiac device. These studies emphasized the importance of early detection of AF for predicting clinical HF. Thus, it is important with early S‐AF detection for preventing HF hospitalization. Catheter ablation for AF and amiodarone might affect the clinical event such as ICD therapy as well as HF hospitalization. However, there was no significant difference with catheter ablation and amiodarone in our study.

### 4.4 Inappropriate therapy induced by S‐AF

Previous study^18^ reported that the rate of inappropriate therapy was 13% in patients without AF, 28% in patients with paroxysmal AF, 18% in patients with persistent AF and 32% in patients with permanent AF. In the no‐AF group, new‐onset AF during follow‐up was the cause of inappropriate device shocks in 27(4%) patients. We described that 4 (13%) in patients with S‐AF had inappropriate therapies, 4 (8.9%) in patients with C‐AF had inappropriate therapies, and 6 (7.7%) in patients without AF had inappropriate therapies. The data by Borleffs et al included patients with a history of AF, on the other hand, our data excluded patients with a history of AF. Other studies^19^, ^20^ demonstrated the relationship between the existence of AF and inappropriate device discharge. In addition, they also reported the consequent negative effects of inappropriate device discharge on patient quality of life and demonstrated the impact of inappropriate shock delivery on mortality. Poole^21^ et al also reported that the occurrence of inappropriate ICD shock was associated with a significant increase in the risk of death as compared with no inappropriate shock. The most common cause of death among patients who received any ICD shock was progressive HF. On the other hand, there was no relationship between AF and mortality in our study. Our data excluded patients with a history of AF and contained only a small number of patients. However, there was a significant relationship between S‐AF and HF hospitalization, and our finding show that inappropriate therapy might affect the HF hospitalization in patients with S‐AF.

## 5 LIMITATIONS

The study has several limitations. First, this study was a retrospective nonrandomized, single‐center study and the decision to implant a CRT device was likely based on multiple factors. Second, this study had a small number of patients, and the results therefore must be interpreted with caution. However, we believe that this study is an adequate evaluation as we found a significant association between S‐AF and HF hospitalization. Third, S‐AF was defined as AF was detected by device report in the absence of symptoms, because symptoms are subjective and are therefore difficult to evaluate. We need a prospective study under the correct definition to certain the relationship between S‐AF and HF hospitalization. Finally, there was the slight difference in the configuration used for AF detection because of the use of different CRT‐D manufacturers. Further studies will be required to be certain the relationship between S‐AF and HF hospitalization.

## 6 CONCLUSIONS

S‐AF after CRT‐D implantation was associated with a significantly increased risk of HF hospitalization. The loss of the biventricular pacing and increasing an inappropriate therapy might affect the risk of HF hospitalization.

## CONFLICT OF INTEREST

The authors declare no potential conflict of interest.

## Data Availability

The data that support the findings of this study are available from the corresponding author upon reasonable request.
